# Synthesis and Antiproliferative Activity of Novel *All-Trans*-Retinoic Acid-Podophyllotoxin Conjugate towards Human Gastric Cancer Cells

**DOI:** 10.3390/molecules22040628

**Published:** 2017-04-17

**Authors:** Lei Zhang, Jing Wang, Lai Liu, Chengyue Zheng, Yang Wang

**Affiliations:** School of Pharmacy, Zunyi Medical University, 201 Dalian Road, Zunyi 563003, Guizhou, China; LL690226@163.com (L.L.); zhengchengyueyue@126.com (C.Z.); Wangjs3089@163.com (Y.W.)

**Keywords:** *all-trans*-retinoic acid, podophyllotoxin, conjugate, human gastric cancer, anticancer activity

## Abstract

With the purpose of creating a multifunctional drug for gastric cancer treatment, a novel *all-trans*-retinoic acid (**ATRA**) conjugate with podophyllotoxin (**PPT**) was designed and synthesized, and its in vitro antiproliferative activity was evaluated against human gastric cancer cell lines using CCK-8 assay. The conjugate, **P-A**, exhibited significant anticancer activity against MKN-45 and BGC-823 cells with IC_50_ values of 0.419 ± 0.032 and 0.202 ± 0.055 μM, respectively. Moreover, **P-A** efficiently triggered cell cycle arrest and induced apoptosis in MKN-45 and BGC-823 cells due to modulation of cell cycle arrest- (CDK1, CDK2, CyclinA and CyclinB1) and apoptosis- (cleaved caspase-3, -8 and -9) related proteins, respectively. Further mechanism studies revealed that **P-A** could increase the expression levels of RARα and RARβ, and decrease the level of RARγ in MKN-45 and BGC-823 cells. Finally, **P-A** inhibited the ERK1/2 and AKT signaling in the above two cancer cell lines. More importantly, the underlying mechanisms of **P-A** were similar to those of precursor **PPT** but different with the other precursor **ATRA**. Together, the conjugate **P-A** was a promising candidate for the potential treatment of human gastric cancer.

## 1. Introduction

Gastric cancer (GC), a common malignancy of the gastric, is the fifth most common type of cancer [1]. Systemic chemotherapy is the main treatment method for GC patients [2]. However, the survival rate remains low with chemotherapy, having toxic side effects [3,4]. Therefore, there is a pressing need to investigate novel anticancer agents against gastric cancer.

**Figure 1:**
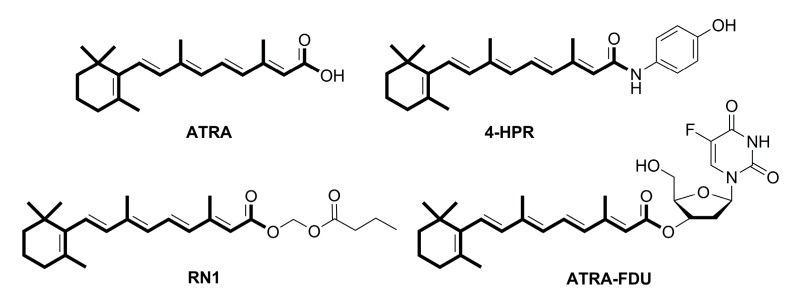
The structures of *all-trans*-retinoic acid and its derivatives.

Retinoic acid (RA), an endogenous metabolite of vitamin A (retinol), is an essential regulator of cell growth and differentiation [5]. RA exists in four isomeric forms, *all-trans* retinoic acid (**ATRA**, Figure 1), 9-*cis*-retinoic acid, 9,13-*dicis*-retinoic acid and 13-*cis*-retinoic acid [6]. Among them, **ATRA** is thought to be the main active isomer displaying its activity by nuclear retinoic acid receptors (RARs), which have three types: α, β and γ, and subsequently regulating gene transcription. **ATRA** regulates many biological processes, such as cell differentiation, reproduction, and regulation of the immune system [7,8,9]. Additionally, RA is connected with the development of several diseases, including neoplasms [5]. So far, the treatment of acute promyelocytic leukemia by **ATRA** has been conducted [10]. However, intolerable side effects and high clearance hinder the clinical application of **ATRA**. To overcome the shortcomings, many chemical modifications of **ATRA** have been prepared, and several important agents, including 4-HPR, RN1 and ATRA-FDU (Figure 1), were reported to have the potential to kill various cancers, such as leukemia, breast cancer, lung cancer, pancreatic cancer, colon cancer and melanoma carcinoma [11,12,13,14,15]. Wu and co-workers showed that both RARα and RARβ were mediators in the antineoplastic function of **ATRA** in gastric MKN-45 cells [16]. Meanwhile, **ATRA** could induce gastric BGC-823 cell death by apoptosis, cell cycle arrest and regulation of RARα [17,18,19].

**Figure 2:**
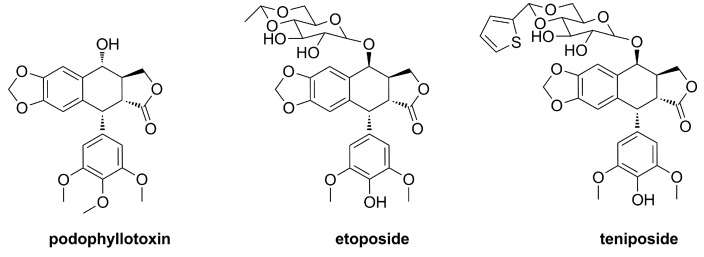
The structures of podophyllotoxin and its derivatives.

It is noteworthy that podophyllotoxin (**PPT**, Figure 2), a naturally-occurring aryltetralin lignan isolated mainly from *Podophyllum* species, shows important antineoplastic properties by inhibition of microtubule assembly [20]. Due to intolerable side effects (e.g., vomiting, diarrhea, paralytic ileus and central nervous system depression), the clinical potential of podophyllotoxin as an anticancer drug has not been reached [21]. In this regard, extensive structural modifications of podophyllotoxin have been performed to improve the pharmacological properties [22], including the well-known and approved drugs etoposide and teniposide (Figure 2), which have been widely used for the clinical chemotherapy of neuroblastoma, testicular cancer, acute leukaemia and Hodgkin's lymphoma. Diverse derivatives like TOP-53, GL-331, NK-611 and Tafluposide are presently under clinical investigations [23]. Recent studies indicated that podophyllotoxin derivatives also exhibited potential antiproliferative activity against gastric cancer, including MKN-45 and BGC-823 cells, by induction of apoptosis [24,25,26].

**Figure 3:**
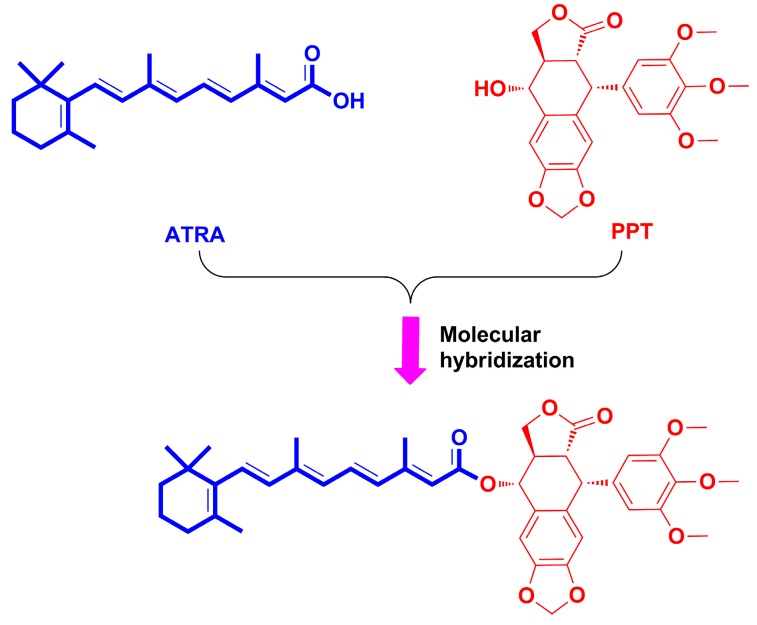
Design of novel *all-trans*-retinoic acid-podophyllotoxin conjugate.

To create more effective antitumor candidates that more affordable, hybridization of natural products or natural product-drug is one promising approach for designing lead compounds [27,28]. Recently, our group has described the synthesis and antiproliferative potential of the **PPT** conjugates [29,30,31,32,33,34,35], and some derivatives showed dramatic activity. For example, the artesunate-podophyllotoxin conjugate exhibited notable cytotoxicity against several cancer cell lines. Previous reports demonstrated that both **ATRA** and **PPT** derivatives displayed antiproliferative activity towards gastric cancer cells. Moreover, the expression of RARs in various gastric cancer cell lines was different, which was connected with the antiproliferative effect of **ATRA**, however, whether the anticancer activity of **PPT** against gastric cancer cells was connected with the RARs remains unclear. In addition, it is also unknown whether the conjugate of **ATRA** and **PPT** exhibits more anti-gastric cancer potential and less toxicity. The present article described the design, synthesis and in vitro antiproliferative activity of a novel conjugate of **ATRA** and **PPT** (Figure 3) using molecular hybridization strategy [36] against two human gastric cancer cell lines (MKN-45 and BGC-823) and a normal human liver cell line (L-O2). The conjugate was preliminarily investigated for its underlying molecular mechanisms. 

## 2. Results and Discussion

### 2.1. Chemistry

**Scheme 1:**
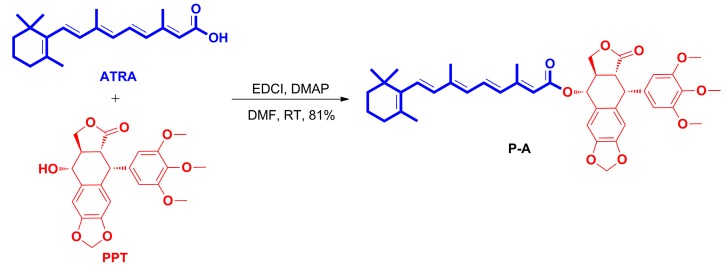
Synthesis of the *all-trans*-retinoic acid-podophyllotoxin conjugate.

The target compound, *all-trans*-retinoic acid-podophyllotoxin conjugate (**P-A**), was synthesized as depicted in Scheme 1. The coupling of *all-trans*-retinoic acid with podophyllotoxin was successfully achieved using 1-(3-dimethylaminopropyl)-3-ethylcarbodiimide hydrochloride (EDCI) and 4-dimethyaminopyridine (DMAP) in dimethylformamide (DMF) at room temperature to give the targeted **P-A**, in yield of 81%. The synthesized conjugate was fully characterized by ^1^H-NMR, ^13^C-NMR and high resolution mass spectrum (HR-MS).

### 2.2. Antiproliferative Activity

The **P-A** conjugate and its precursors (**PPT** and **ATRA**) as well as a reference anticancer drug called etoposide, were initially evaluated by CCK-8 assay for their in vitro antiproliferative effects on human gastric cancer cell lines (MKN-45 and BGC-823) and a normal human liver cell line (L-O2). The IC_50_ ± SD values of the tested molecules are presented in Table 1. In general, conjugate **P-A** possessed significant antiproliferative activity toward two human gastric cancer cell lines (MKN-45 and BGC-823) with IC_50_ values of 0.419 ± 0.032 and 0.202 ± 0.055 μM, respectively, which were much stronger than those of **ATRA** and etoposide, respectively, but less than **PPT**. Meanwhile, precursor **ATRA** displayed weak antiproliferative activities toward above two cancer cell lines with IC_50_ values of 88.462 ± 6.931 and 83.076 ± 15.252 μM, respectively, while the IC_50_ values of the control drug etoposide were 2.88 ± 0.532 and 1.737 ± 0.294 μM, respectively. In addition, precursor **PPT** showed higher antiproliferative activities against MKN-45 and BGC-823 with IC_50_ values of 0.045 ± 0.012 and 0.03 ± 0.005 μM, respectively, however, it also exhibited the most potent cytotoxic activity toward the normal human liver cell line L-O2 with an IC_50_ value of 0.037 ± 0.008 μM. In contrast, conjugate **P-A** showed less antiproliferative effect on L-O2 cells than that of **PPT**. Our data showed that **P-A** was a potential cytotoxic agent, and although its cytotoxicity was less than that of the parent compound **PPT**, which may be associated with the solubility property, it improved on that of **ATRA**.

### 2.3. Cell Cycle Analysis

**Figure 4:**
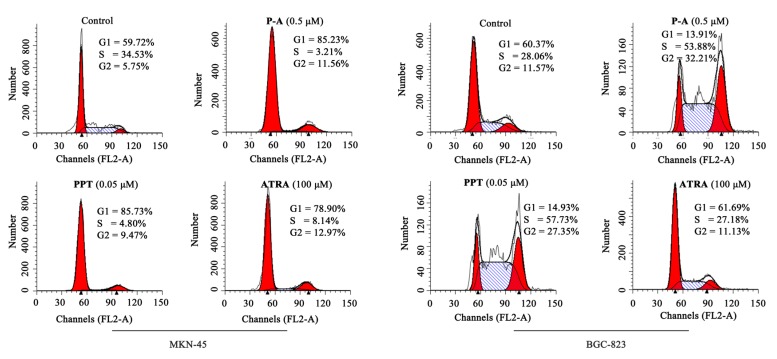
Effects of **P-A**, podophyllotoxin **(PPT)** and the *all-trans*-retinoic acid **(ATRA)** on the cell cycle of MKN-45 and BGC-823 cells. Cells were incubated with 1% DMSO, **P-A** (0.5 μM), **PPT** (0.05 μM) or **ATRA** (100 μM) for 48 h.

To determine the effect of conjugate **P-A** on the cell cycle, MKN-45 and BGC-823 cells were incubated with different concentrations of **P-A**, **PPT** or **ATRA** for 48 h and then assessed by flow cytometry. Results are shown in Figure 4. Treatment with **P-A** (0.5 μM), **PPT** (0.05 μM) or **ATRA** (100 μM) could arrest MKN-45 cells at G1 phase. However, incubation with **P-A** (0.5 μM) and **PPT** (0.05 μM) significantly blocked BGC-823 cells at the G2 phase, while **ATRA** (100 μM) did not show significant effect on the cycle of BGC-823 cells. Additionally, as shown in Figure 4, after treatment with 0.5 μM **P-A**, 0.05 μM **PPT** and 100 μM **ATRA**, 85.23%, 85.73% and 78.90%, respectively, cell numbers (MKN-45) were accumulated at the G1 phase. In contrast, the percentages of BGC-823 cells at S phase were 53.88% and 57.73% in **P-A** (0.5 μM) and **PPT** (0.05 μM)-treated groups, respectively. Taken together, the data showed that conjugate **P-A** blocked MKN-45 cells at G1 phase, but induced BGC-823 cells at S phase.

### 2.4. Cell Apoptosis Analysis

**Figure 5:**
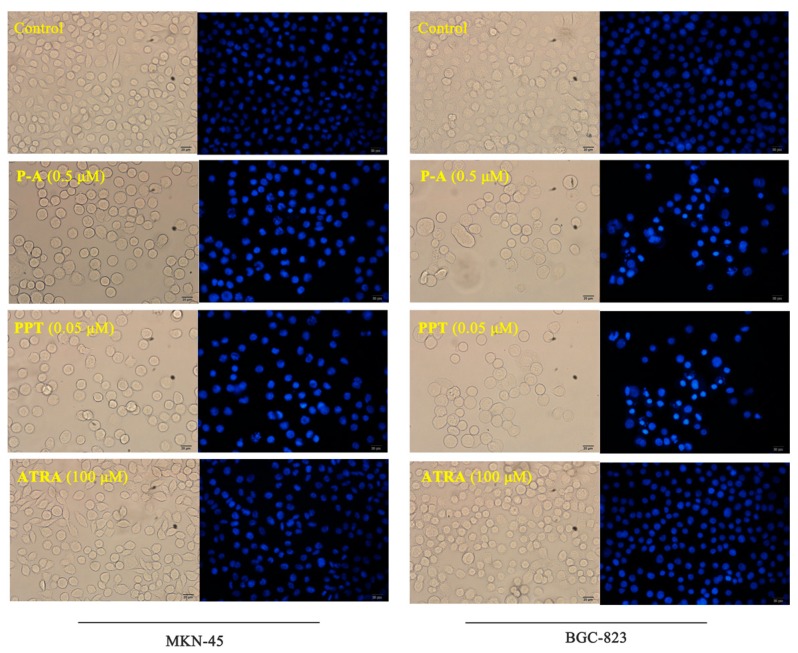
Effects of **P-A**, **PPT** and **ATRA** on the morphology of MKN-45 and BGC-823 cells. Cells were incubated with 1% DMSO, **P-A** (0.5 μM), **PPT** (0.05 μM) or **ATRA** (100 μM) for 24 h. Subsequently, cells were stained with Hoechst 33342 for microscopy. Magnification, ×200; scale, 20 μm.

**Figure 6:**
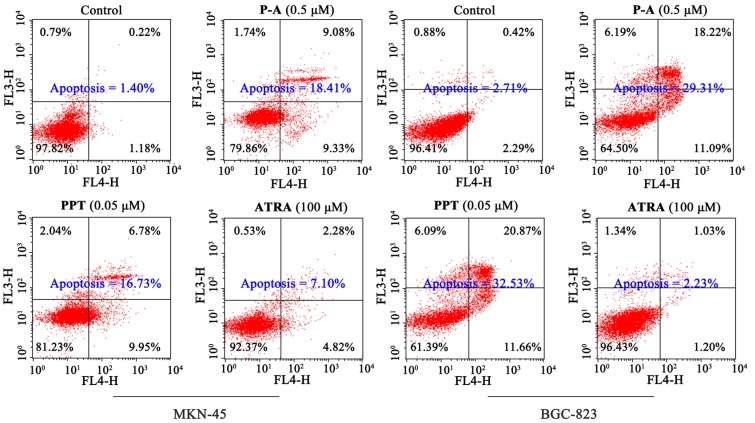
Effects of **P-A**, **PPT** and **ATRA** on the apoptosis of MKN-45 and BGC-823 cells. Cells were incubated with 1% DMSO, **P-A** (0.5 μM), **PPT** (0.05 μM) or **ATRA** (100 μM) for 48 h.

Subsequently, we tested the effect of conjugate **P-A** on the cell apoptosis by Hoechst 33342 staining and flow cytometry. As shown in Figure 5, condensation of chromatin, as well as bright fluorescent in apoptosis cells were observed after MKN-45 and BGC-823 cells were treated with **P-A** (0.5 μM) and **PPT** (0.05 μM), respectively. However, no noticeable effects were observed in the **ATRA** (100 μM) group in two gastric cancer cell lines. Furthermore, Figure 6 shows that the apoptotic cell number increased to 18.41%, 16.73% and 7.10% when MKN-45 cells were incubated with **P-A** (0.5 μM), **PPT** (0.05 μM) and **ATRA** (100 μM), respectively, compared to 1.40% in the control group. Apoptotic cell number increased to 29.31% and 32.53% when BGC-823 cells were treated with **P-A** (0.5 μM) and **PPT** (0.05 μM), respectively, and **ATRA** (100 μM) showed no significant effect. These data demonstrated that conjugate **P-A** significantly induced both MKN-45 and BGC-823 cells apoptosis, and that BGC-823 cells were more sensitive than MKN-45 cells to **P-A**.

### 2.5. Expression of Cell Cycle-Related Proteins

**Figure 7:**
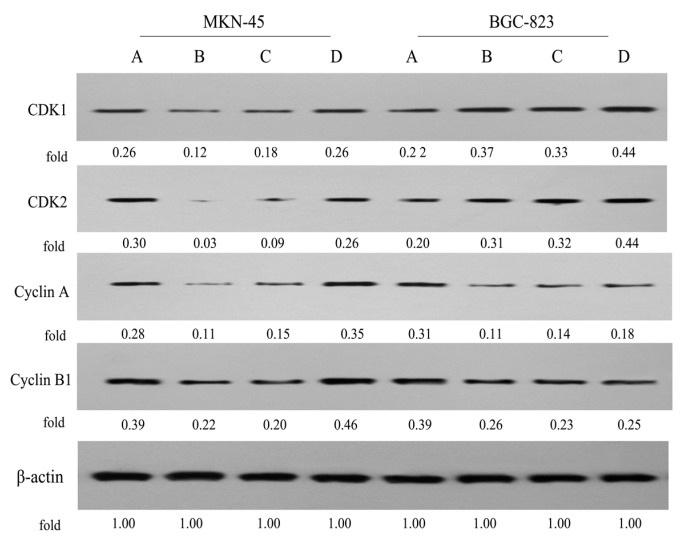
Effects of **P-A**, **PPT** and **ATRA** on the expression levels of cell cycle-related proteins in MKN-45 and BGC-823 cells by western blotting using β-actin as control. (**A**) Control cells; (**B**) Cells treated with **P-A** (0.5 μM); (**C**) Cells treated with **PPT** (0.05 μM); (**D**) Cells treated with **ATRA** (100 μM).

Furthermore, we investigated the impact of conjugate **P-A** treatment on the expression of cell cycle-related proteins in MKN-45 and BGC-823 cells by Western blot, as shown in Figure 7. In MKN-45 cells, treatment with **P-A** (0.5 μM) and **PPT** (0.05 μM) significantly decreased the levels of CDK1, CDK2, CyclinA and CyclinB1, respectively. Simultaneously, **ATRA** (100 μM) only slightly increased the expression levels of CyclinA and CyclinB1. Additionally, in BGC-823 cells, **P-A** (0.5 μM), **PPT** (0.05 μM) and **ATRA** (100 μM) could upregulate the levels of CDK1 and CDK2, but suppressed the levels of CyclinA and CyclinB1. Hence, our data indicated that hybrid molecule **P-A** showed different mechanisms underlying the cell cycle arrest in gastric MKN-45 and BGC-823 cells, which may explain why **P-A** blocked MKN-45 cells at G1 phase, but induced BGC-823 cells at S phase in flow cytometry.

### 2.6. Expression of Apoptosis-Related Proteins

**Figure 8:**
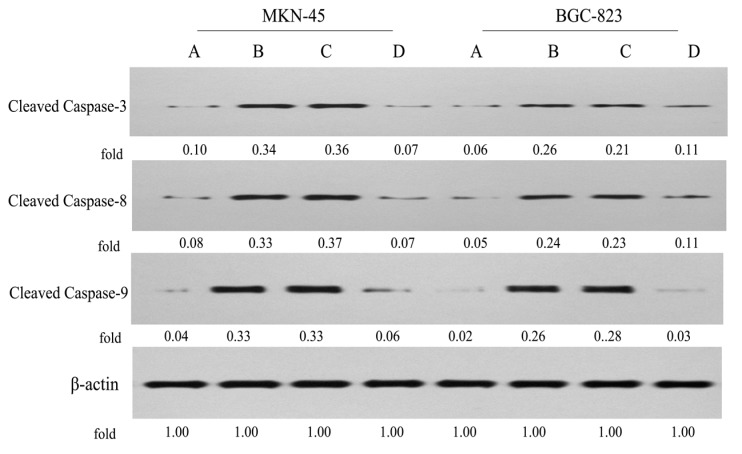
Effects of **P-A**, **PPT** and **ATRA** on the expression levels of apoptosis-related proteins in MKN-45 and BGC-823 cells by western blotting using β-actin as control. (**A**) Control cells; (**B**) Cells treated with **P-A** (0.5 μM); (**C**) Cells treated with **PPT** (0.05 μM); (**D**) Cells treated with **ATRA** (100 μM).

To further validate the underlying mechanisms of apoptosis induced by conjugate **P-A**, the expression levels of cleaved caspase-3, -8 and -9 in MKN-45 and BGC-823 cells were examined by Western blot (Figure 8). We observed that **P-A** (0.5 μM) and **PPT** (0.05 μM) significantly enhanced the levels of cleaved caspase-3, -8 and -9 in both MKN-45 and BGC-823 cells, compared to the control group. However, **ATRA** (100 μM) displayed no significant effect on MKN-45 cells, but it slightly increased the levels of cleaved caspase-3 and -8 in BGC-823 cells. These results revealed that conjugate **P-A** induced apoptosis in MKN-45 and BGC-823 cells through stimulation of intrinsic and external mitochondrial pathways.

### 2.7. Expression of RARs

**Figure 9:**
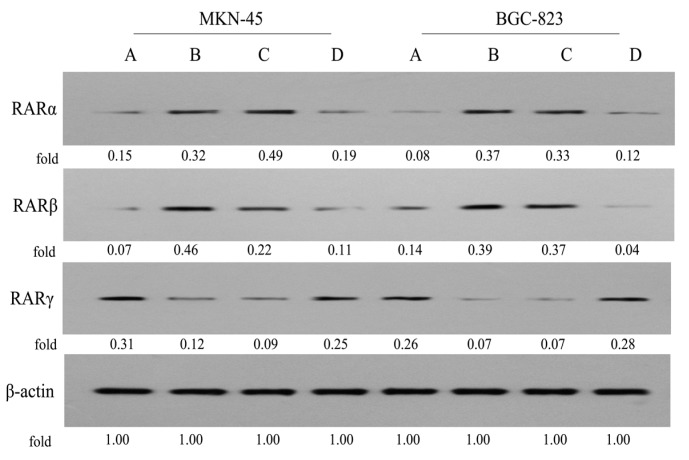
Effects of **P-A**, **PPT** and **ATRA** on the expression levels of RARs in MKN-45 and BGC-823 cells by western blotting using β-actin as control. (**A**) Control cells; (**B**) Cells treated with **P-A** (0.5 μM); (**C**) Cells treated with **PPT** (0.05 μM); (**D**) Cells treated with **ATRA** (100 μM).

It was reported that RARs were mediators in the anticancer function of **ATRA** in gastric MKN-45 and BGC-823 cells [16,17,18,19]. However, to the authors’ knowledge, little is known about the RARs regarding the involvement of **PPT** in gastric carcinoma. In order to gain more insight into the mechanisms of conjugate **P-A** in suppressing gastric cancer cell proliferation, we then examined the levels of RARs in MKN-45 and BGC-823 cells using Western blot assay. As seen in Figure 9, initially we found that the expression level of RARα was higher, but the level of RARβ was lower in MKN-45 cells, compared to in BGC-823 cells. Also, the RARγ level in both two cancer cell lines was almost equipotent. When MKN-45 and BGC-823 cells were treated with **P-A** (0.5 μM), the expression levels of RARα and RARβ were increased, while the level of RARγ was decreased, which may be associated with the **PPT** skeleton, because 0.05 μM **PPT** could stimulate the expression of RARα and RARβ, but inhibit the expression of RARγ. Moreover, **ATRA** (100 μM) only slightly increased RARα and RARβ levels, but decreased RARγ level in MKN-45 cells. In addition, **ATRA** (100 μM) slightly increased the level of RARα, downregulated RARβ expression, and had no notable effect on the level of RARγ in BGC-823 cells. These data indicated that **PPT** could regulate the expression levels of RARs in gastric cancer cells for the first time, as well as the conjugate **P-A**. Similar to its precursor **PPT**, it also modulated RARs levels in both MKN-45 and BGC-823 cells, which were different from another precursor, **ATRA**.

### 2.8. Effects on the ERK1/2, STAT3, mTOR and AKT Signaling

**Figure 10:**
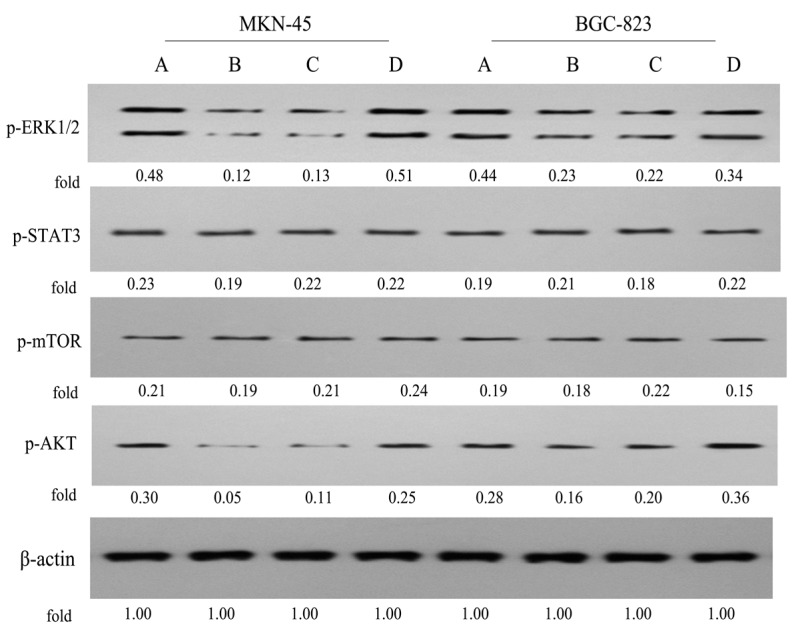
Effects of **P-A**, **PPT** and **ATRA** on the ERK1/2, STAT3, mTOR and AKT signaling in MKN-45 and BGC-823 cells by western blotting using β-actin as control. (**A**) Control cells; (**B**) Cells treated with **P-A** (0.5 μM); (**C**) Cells treated with **PPT** (0.05 μM); (**D**) Cells treated with **ATRA** (100 μM).

Recent studies demonstrated that some signalling, such as ERK1/2, STAT3, mTOR and AKT, were closely related to the development of carcinoma [37,38]. To further investigate the precise mechanisms of **P-A**, we examined the regulative effects of **P-A** on the ERK1/2, AKT, mTOR and STAT3 signalling in MKN-45 and BGC-823 cells (Figure 10). It was found that **P-A** (0.5 μM) and **PPT** (0.05 μM) significantly suppressed the phosphorylations of ERK1/2 and AKT in MKN-45 and BGC-823 cells, respectively, and they did not alter the phosphorylation of STAT3 and mTOR. Conversely, **ATRA** (100 μM) only slightly blocked the phosphorylation of AKT in MKN-45 cells, and it increased the phosphorylation of AKT and restrained the phosphorylation of ERK1/2 and mTOR in BGC-823 cells. These results proved that the conjugate **P-A** could notably inhibit the ERK1/2 and AKT signalling in MKN-45 and BGC-823 cells, which was similar to precursor **PPT**, but different from the other precursor **ATRA**.

## 3. Materials and Methods

### 3.1. Chemistry

#### 3.1.1. General

Melting point was determined on an electric SGWX-4 digital visual melting point apparatus. The ^1^H-NMR and ^13^C-NMR spectra were acquired on Agilent instrument (400 MHz), using TMS as an internal standard. HRMS was obtained on Agilent 6520 TOP-MS instrument. Column chromatography was performed on flash silica gel (200–300 mesh). Reaction was monitored by TLC on silica gel, and detected by UV light (254 nm) accordingly.

#### 3.1.2. Preparation of *All-Trans*-Retinoic Acid-Podophyllotoxin Conjugate **P-A**

To a stirred solution of podophyllotoxin (0.29 mmol), all-trans-retinoic acid (0.23 mmol) and 4-dimethylaminopyridine (DMAP) (0.34 mmol) in *N*,*N*-dimethylformamide (DMF) (4 mL) was added 1-(3-dimethylaminopropyl)-3-ethylcarbodiimide hydrochloride (EDCI) (0.57 mmol) at 0 °C. The mixture was stirred at 0 °C for 10 min and then for a further 6 h at room temperature (RT = 25 °C). Reaction solution was poured into water (40 mL) and the residue was collected by filtration and dried in vacuum oven. The crude was purified by column chromatography (ethyl acetate/petroleum ether = 1:4) to obtain the *all-trans*-retinoic acid-podophyllotoxin conjugate (**P-A**).

Compound **P-A**: *R*_f_ = 0.51 (ethyl acetate/petroleum ether = 1:4), yellow powder, yield 81%; m.p.: 102–104 °C; ^1^H-NMR (400 MHz, CDCl_3_) δ 7.08 (t, *J* = 12.0 Hz, 1H, polyene chain), 6.82 (s, 1H, Ar-H), 6.53 (s, 1H, Ar-H), 6.40 (s, 2H, Ar-H), 6.26–6.37 (m, 2H, polyene chain), 6.12–6.16 (m, 2H, polyene chain), 5.97 (d, *J* = 4.0 Hz, 2H, O-CH_2_-O), 5.91 (d, *J* = 8.8 Hz, 1H, Ar-H), 5.81 (s, 1H, polyene chain), 4.61 (d, *J* = 4.0 Hz, 1H, CH-Ar), 4.42 (t, *J* = 7.2 Hz, 1H, CH-*CH*_2_-O), 4.25 (t, *J* = 10.0 Hz, 1H, CH-*CH*_2_-O), 3.80 (s, 3H, OCH_3_), 3.76 (s, 6H, 2 × OCH_3_), 2.93–2.97 (m, 2H, *CH*-CH_2_-O, O=C-CH), 2.38 (s, 3H, CH_3_), 2.01 (s, 5H, CH_2_ in cyclohexene ring, CH_3_), 1.71 (s, 3H, CH_3_), 1.58–1.62 (m, 2H, CH_2_ in cyclohexene ring), 1.44–1.47 (m, 2H, CH_2_ in cyclohexene ring), 1.02 (s, 6H, 2 × CH_3_); ^13^C-NMR (100 MHz, CDCl_3_) δ 173.93, 162.57, 155.10, 152.54, 147.95, 147.50, 140.58, 137.09, 134.98, 132.17, 129.26, 116.84, 109,60, 107,94, 107.19, 101.52, 72.64, 71.65, 60.75, 56.09, 45.63, 43.76, 39.54, 34.24, 33.10, 31.45, 28.95, 27.26, 21.77, 19.17, 14.08, 13.23, 12.98; HRMS-ESI (*m*/*z*): calcd. for C_42_H_52_NO_9_ [M + NH_4_]^+^ 714.3637, found 714.3633.

### 3.2. Biology

#### 3.2.1. Cytotoxicity Assays

Antiproliferative assay was performed on human gastric cancer cells (MKN-45 and BGC-823) and normal human liver cells (L-O2). Cells were seeded into 96-well micro test plates. After 72 h of incubation with tested compounds at various concentrations at 37 °C in a humidified atmosphere with 5% CO_2_, CCK-8 (10 μL) was added to each well and cells were incubated for 2 h at 37 °C. Absorbance of each solution was read at 450 nm wavelength. The IC_50_ value of each tested compound was calculated by software SPSS.

#### 3.2.2. Cell Cycle Analysis

MKN-45 and BGC-823 cells were plated in 6-well plates and incubated with tested compounds for 48 h at 37 °C and 5% CO_2_, respectively. Next, cells were washed with PBS, collected, and fixed with cold 70% ethanol at 4 °C overnight, then treated with RNase for 30 min at 37 °C, and stained with PI for 30 min in dark. The percentages of cells were determined using a FACScan flow cytometer.

#### 3.2.3. Hoechst 33242 Staining

MKN-45 and BGC-823 cells were incubated with tested compounds for 24 h at 37 °C and 5% CO_2_, respectively. After incubating, the cells were washed with PBS two times and incubated for 10 min with Hoechst 33342 staining buffer in dark. Cells were then analyzed by inverted microscope and fluorescent microscope.

#### 3.2.4. Cell Apoptosis Analysis

MKN-45 and BGC-823 cells were seeded in 6-well plates and then treated with tested compounds for 48 h at 37 °C and 5% CO_2_, respectively. Next, cells were collected and washed twice with, and then stained with 5 μL Annexin V-APC and 5 μL 7-AAD at room temperature for 15 min in the dark. Apoptotic cells were detected using a FACScan flow cytometer.

#### 3.2.5. Western Blot Analysis

MKN-45 and BGC-823 cells were incubated with tested compounds for 48 h at 37 °C and 5% CO_2_, respectively, and then cells were harvested, washed and lysed in complete lysis buffer. The concentration of protein was determined by bicinchonininc acid (BCA) assay kit. Protein samples were separated by 10% sodium dodecyl sulfate-polyacrylamide gel electro-phoresis (SDS-PAGE). Next, the proteins were transferred to a nitrocellulose membrane, blocked for 2 h with 5% skimmed milk at room temperature. Membrane was incubated overnight at 4 °C with primary antibodies: CDK1, CDK2, CyclinA, CyclinB1, cleaved caspase-3, cleaved caspase-8, cleaved caspase-9, RARα, RARβ, RARγ, p-ERK1/2, p-STAT3, p-AKT, p-mTOR and β-actin. Unbound antibody was washed with TBST for 15 min and membrane was incubated with the secondary antibody at room temperature for 2 h. Finally, membrane was detected by chemiluminescence detection system.

## 4. Conclusions

In conclusion, the conjugate **P-A** obtained in the esterification reaction between the well-known anticancer compound *all-trans*-retinoic acid and natural compound podophyllotoxin displayed interesting antiproliferative activity in two human gastric cancer cells lines at nanomole concentration. The anticancer effect of **P-A** was also closely connected with cell cycle arrest and apoptosis by modulating the cell cycle arrest- and apoptosis-related proteins expression levels, as well as the levels of RARs. Moreover, the conjugate **P-A** could block the ERK1/2 and AKT signalling in two gastric cancer cells. Our data suggested that the antineoplastic effect of the conjugate **P-A** may be mainly due to precursor **PPT**. As topoisomerase II is the mechanism of podophyllotoxin, it is also important to know if the hybrid it holds the same mechanism. Further study on the topoisomerase II inhibition and other molecular mechanisms of **P-A** will be carried out in due course. Overall, these results indicated that *all-trans*-retinoic acid-podophyllotoxin conjugate **P-A** had potent antiproliferative activity against human gastric cancer.

## Figures and Tables

**Table 1:** Antiproliferative activity of tested compounds.

Compound	IC_50_ (μM) ^1,2^		
	MKN-45	BGC-823	L-O2
**P-A**	0.419 ± 0.032	0.202 ± 0.055	0.293 ± 0.081
**PPT**	0.045 ± 0.012	0.03 ± 0.005	0.037 ± 0.008
**ATRA**	88.462 ± 6.931	83.076 ± 15.252	>100
Etoposide	2.88 ± 0.532	1.737 ± 0.294	2.006 ± 0.24

^1^ CCK-8 methods, drug exposure was for 72 h; ^2^ Data were expressed as mean IC_50_ ± SD from three independent experiments.

## Supplementary Materials

Supplementary materials are available online.
