# Supplementary material for: Synthesis and Antiproliferative Activity of Novel All-Trans-Retinoic Acid-Podophyllotoxin Conjugate towards Human Gastric Cancer Cells
Source: Molecules. 2017 Apr 17;22(4):628. doi: 10.3390/molecules22040628 (PMC6154554; doi:10.3390/molecules22040628)
Supplement: Supplementary file 1 [file molecules-22-00628-s001.pdf]

Supplementary Materials:

Synthesis and Antiproliferative Activity of Novel *All-Trans*-Retinoic Acid-Podophyllotoxin Conjugate towards Human Gastric Cancer Cells

Lei Zhang *, Jing Wang *, Lai Liu , Chengyue Zheng and Yang Wang

P-A HR-MS

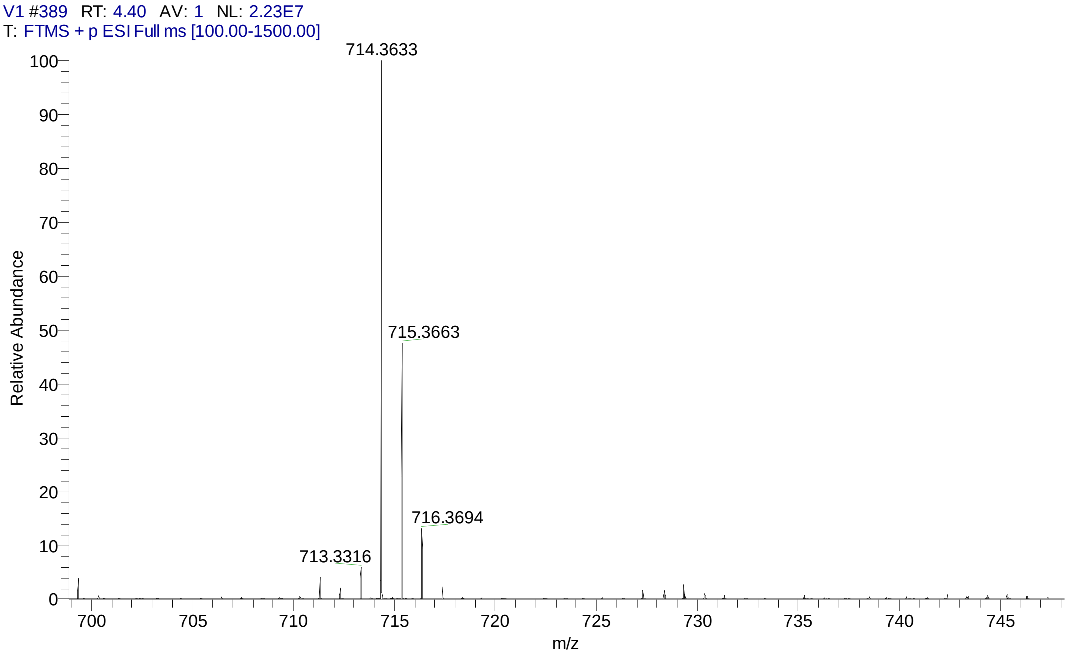

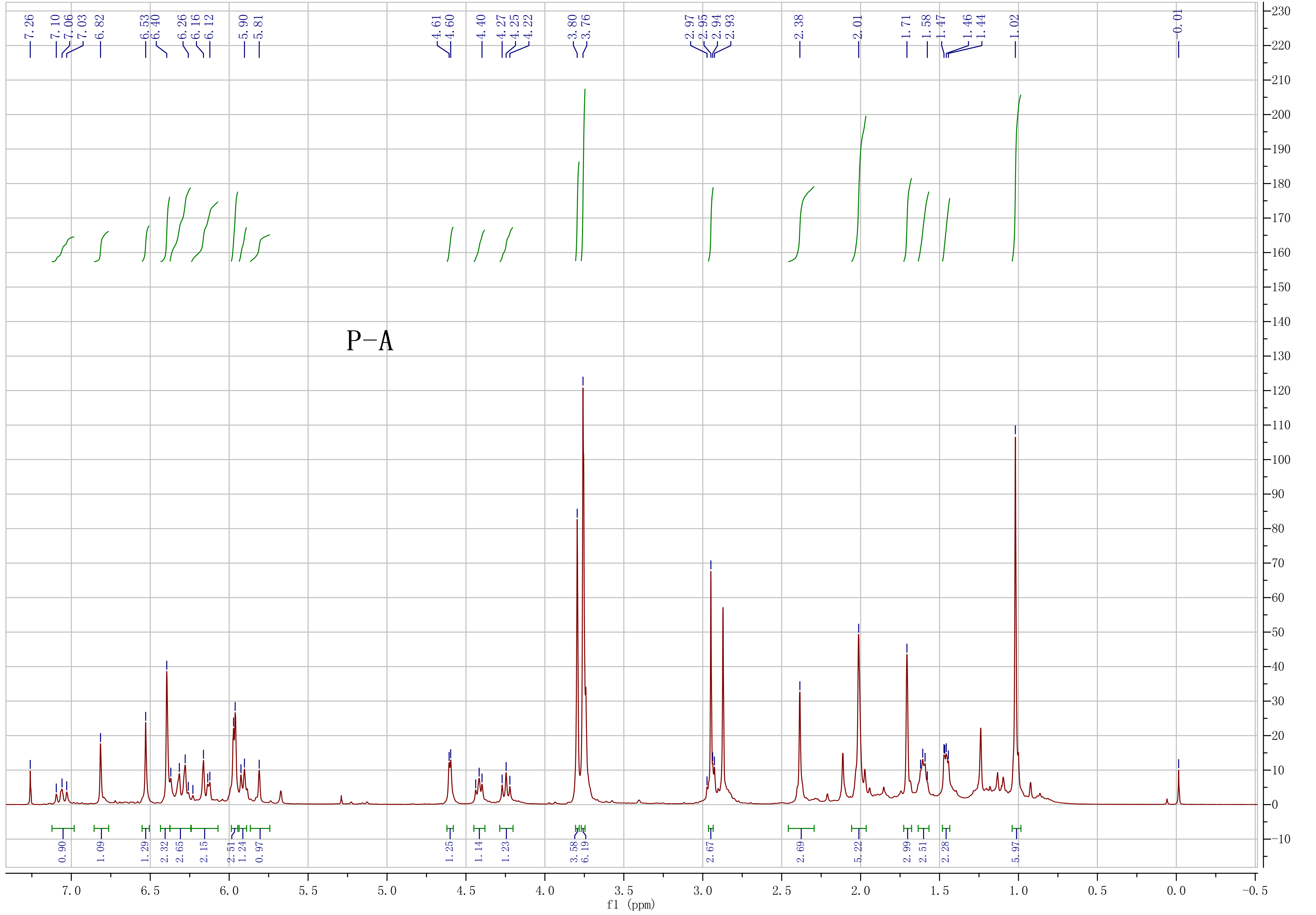

P-A
7.26
7.10
7.06
7.03
6.82
6.53
6.40
6.26
6.16
6.12
5.90
5.81
4.61
4.60
4.40
4.27
4.25
4.22
3.80
3.76
2.97
2.95
2.94
2.93
2.38
2.01
1.71
1.58
1.47
1.46
1.44
1.02
-0.01
0.90
1.09
1.29
2.32
2.65
2.15
2.51
1.24
0.97
1.25
1.14
1.23
3.58
6.19
2.67
2.69
5.22
2.99
2.51
2.28
5.97
f1 (ppm)

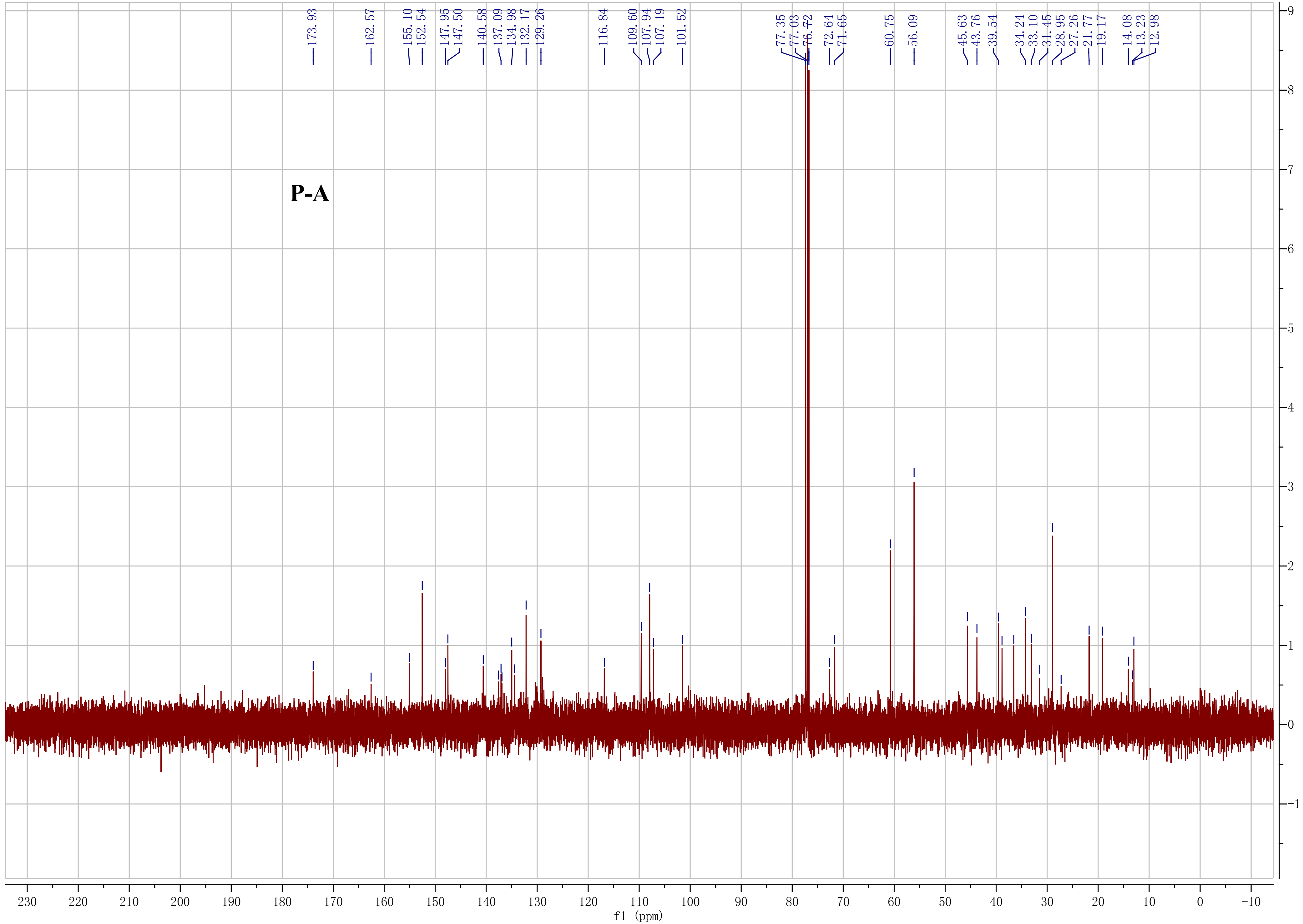
P-A
173.93
162.57
155.10
152.54
147.95
147.50
140.58
137.09
134.98
132.17
129.26
116.84
109.60
107.94
107.19
101.52
77.35
77.03
76.72
72.64
71.65
60.75
56.09
45.63
43.76
39.54
34.24
33.10
31.45
28.95
27.26
21.77
19.17
14.08
13.23
12.98
f1 (ppm)
